# Metacognitive Beliefs as Predictors of Return to Work After Intensive Return-to-Work Rehabilitation in Patients With Chronic Pain, Chronic Fatigue and Common Psychological Disorders: Results From a Prospective Trial

**DOI:** 10.3389/fpsyg.2020.00070

**Published:** 2020-02-06

**Authors:** Henrik B. Jacobsen, Mari Glette, Karen W. Hara, Tore C. Stiles

**Affiliations:** ^1^Department of Pain Management and Research, Division of Emergencies and Critical Care, Oslo University Hospital, Oslo, Norway; ^2^Department of Psychology, Faculty of Social Sciences, University of Oslo, Oslo, Norway; ^3^CatoSenteret Rehabilitation Center, Son, Norway; ^4^Department of Circulation and Medical Imaging, Faculty of Medicine and Health Sciences, Norwegian University of Science and Technology, Trondheim, Norway; ^5^Department of Public Health and Nursing, Faculty of Medicine and Health Sciences, Norwegian University of Science and Technology, Trondheim, Norway; ^6^Norwegian Labour and Welfare Administration, Oslo, Norway; ^7^Department of Psychology, Faculty of Social and Educational Sciences, Norwegian University of Science and Technology, Trondheim, Norway

**Keywords:** rehabilitation, return-to-work, metacognition, prospective, pain, fatigue syndromes

## Abstract

**Background:**

Metacognitions are associated with work status, but no research has examined to what extent metacognitions before treatment and change in metacognitions following treatment predict return to work (RTW) prospectively. The present study aims to address these two gaps in knowledge.

**Methods:**

212 patients on long-term sick leave (>8 weeks) with extensive fatigue, chronic pain conditions and/or mental distress received 3.5 weeks of intensive rehabilitation treatment, aimed at returning them to work. Only part of the population (*n* = 137) had complete follow-up data on metacognitions. Metacognitions were measured with the Metacognitions Questionnaire 30 (MCQ-30), while RTW was measured using official registry data from the Norwegian Labor and Welfare Service. A registry record of participation in competitive work ≥2.5 days (50% work participation) per week, averaging over 14 weeks, was chosen as an outcome reflecting a successful RTW. The registry data spanned a total of 56 weeks per participant.

**Results:**

Our results indicated that baseline MCQ scores was not associated with RTW. This was analyzed for the total MCQ score as well as for all subscales. We observed substantial changes in metacognitions following treatment, and a 1-point change in the total sum of metacognitive beliefs was associated with 5% greater odds for successful RTW at all time points (*p* = 0.040), while a 1-point change on the subscale of beliefs about the need to control thoughts gave 20% greater odds for successful RTW (*p* = 0.016).

**Conclusion:**

Metacognitions concerning the need to control thoughts appear to have a significant influence on patients return to work. Here, we observed that a change in these beliefs following treatment substantially affected RTW over the course of 1 year.

## Introduction

In recent years the metacognitive model has been associated with work participation and absence (Nordahl and Wells, 2017a, b), as well as changes in common mental disorders (Solem et al., 2009; Wells et al., 2012). Depression, anxiety, persistent pain, and fatigue are common justifications for long-term sick leave in Norway (Jacobsen et al., 2015), and most other western countries (Henderson et al., 2005).

However, clinical and epidemiological studies highlight that there is considerable comorbidity amongst anxiety, depression, chronic pain, and fatigue (Kessler et al., 2007; Reme et al., 2011; Jacobsen et al., 2015). This overlap is supported by recent data where the specific reasons justifying sick leave vary, but clinical symptomatology and disorders overlap significantly (Jacobsen et al., 2015; Hara et al., 2017b).

Return to work (RTW) rehabilitation using psychological interventions has been somewhat successful for both musculoskeletal disorders and common mental health disorders. A recent meta-analysis showed a small effect size when psychological rehabilitation is compared to a “treatment as usual” condition (*g* = 0.16) (Finnes et al., 2019). This effect size was similar regardless of diagnoses justifying sick leave and different psychological interventions (Finnes et al., 2019), lending support to interventions targeting transdiagnostic processes of change (Loisel and Anema, 2013; Hara et al., 2017b).

A transdiagnostic stance that may further our understanding of factors that may implicate individuals RTW is the metacognitive model (Wells and Matthews, 1994, 1996).

According to the metacognitive model, psychological distress and emotional disorders are maintained by the activation of a maladaptive thinking style called the cognitive attentional syndrome (CAS). The CAS is characterized by repetitive negative thinking in the form of rumination and worry, and is associated with increased self-focused attention and maladaptive coping behaviors. The CAS is maintained by individual’s metacognitive beliefs, which can be broken down into positive and negative metacognitive beliefs. Positive metacognitive beliefs concern the usefulness of worry (e.g., If I worry I will be prepared), while negative metacognitive beliefs concern the uncontrollability and dangerousness of worry (i.e., worrying could make me lose control) (Wells and Matthews, 1994, 1996).

Recently, studies have begun to evaluate the influence of the metacognitive model on RTW (Nordahl and Wells, 2017a, b). Nordahl and Wells (2017b) investigated the cross-sectional association of metacognitive beliefs and work status in individuals with social anxiety disorder. They found that greater negative metacognitive beliefs were associated with individuals being out of work. More specifically, beliefs regarding the need to control thoughts were greater in those who were out of work.

More broadly, Nordahl and Wells (2017a) evaluated if metacognitive beliefs could predict work status. After controlling for gender, presence of a diagnosed mental health disorder, and trait anxiety (vulnerability to emotional disorder), they found that metacognitive beliefs regarding the need for mental control was a significant predictor of work status over and above the presence of a mental health disorder, and emotional vulnerability. Nordahl and Wells (2017a) highlight that metacognitive beliefs regarding the need to control may lead to increased worrying, threat monitoring, and attempts to control thoughts, which likely decreases cognitive processing capacity for work and impact on individuals interpretations of their ability to work effectively (Nordahl and Wells, 2017a).

Coping strategies might play a significant role in terms of understanding the sick leave process over time. People suffering from depression and anxiety tend to improve symptoms or work-related functioning in the short-term if pushed toward work, but they are vulnerable for falling out again due to anxiety (Knudsen et al., 2013; Oyeflaten et al., 2014). The development of the CAS might play a role in this cyclical pattern of stress and sick leave. Repetitive negative thinking has been shown to delay homeostatic recovery following recovery from induced stress (Capobianco et al., 2018). Similarly, Jacobsen et al. (2014) found that Norwegians on sick leave had a dysregulated stress response in response to an induced stressor. A dysregulated stress response when faced with psychosocial stressors has been associated with depression, anxiety and pain (Kudielka et al., 2007), and is considered by many as a hallmark of chronic fatigue (Wyller et al., 2009).

However, a controversial finding within the field of RTW is the lack of a substantial relationship between symptom levels and work participation (Henderson et al., 2005). However, strong associations have been found between a long duration of depression and work disability (Lagerveld et al., 2010), moreover lifestyle factors affected by symptom severity have also been documented, which again could affect work participation (Blank et al., 2008). Metacognitions can play a crucial role in the resurgence of symptoms, but their relation to RTW has only been investigated cross-sectionally (Nordahl and Wells, 2017a, b). Thus, longitudinal studies are highly warranted (Myhre et al., 2014).

This study aimed to investigate the influence of metacognitions on RTW in a population on long-term sick leave with chronic pain, chronic fatigue and common psychological disorders. RTW was measured over the course of 56 weeks following completion of a common, on-site occupational rehabilitation program. As such we aimed to evaluate: (1) if self-reported metacognitions at baseline are associated RTW in a 12-month period in patients attending an occupational rehabilitation program, (2) if changes in self-reported metacognitions from baseline to time of discharge of the rehabilitation program are associated with long-term RTW.

## Materials and Methods

The current study was an explorative analysis nested within a randomized controlled trial investigating the effectiveness of telephone-guided follow-up versus standard RTW follow-up after on-site occupational rehabilitation. The overarching study is registered in ClinicalTrials.gov (No. NCT01568970).

Project participants, design and flow have been detailed in previous publications (Hara et al., 2017a, b, 2018), as such the subsequent paragraphs provide a brief overview of the project.

### Participants

Participants were referred by general practitioners (GPs) or other medical specialists to a 3.5-week intensive, inpatient rehabilitation from January 2012 to June 2013. The RTW rehabilitation took place at Hysnes Rehabilitation Centre located in the county of Trøndelag, Norway. Upon inclusion participants were invited to take part in the aforementioned study of boosted follow-up. The boosted follow-up consisted of six phone calls from their RTW-coordinator where they discussed progression toward work. Prior to inclusion the participants were assessed by an interdisciplinary team consisting of a physician, psychologist and a physical therapist. Participants completed a comprehensive questionnaire at baseline prior to their first meeting with the assessment team, following which informed consent was obtained and the data from the baseline questionnaire was made available to the researchers.

### Eligibility and Exclusion Criteria

Participants were eligible for the study if referred for either/or persistent pain, fatigue, depression or anxiety to inpatient rehabilitation, participants had to be between 18 and 59 years of age and had to have a clearly stated goal of wanting to RTW. In addition, they had to receive temporary medical benefits due to work incapacity (duration over 8 weeks, partial or full-time). In Norway this involves being on one of two benefits that both require sickness certification; either sickness benefit (compensates for loss of income for employees or others with equivalent rights earned through previous participation in paid work) or work assessment allowance (for those who have either already received sickness benefits for the maximum period of 52 weeks, or have not earned the right to sickness benefits through previous employment).

Participants were to state a self-defined goal of increasing participation in competitive work, be adequately treated for health problems demanding acute care, be able to communicate in Norwegian and to maintain basic daily care for themselves during a stay at the rehabilitation centre. Participants were excluded from the study if they suffered from ongoing mania, psychosis or suicidal ideation, active substance abuse and addiction. Or if they reported pregnancy, planning to enter/return to studies rather than competitive work, incomplete study registration procedure, not registered as receiving temporary medical benefits, or not completing the rehabilitation program due to acute injury/disease or personal/family reasons.

### Study Setting

The 3.5-week inpatient occupational rehabilitation program consisted of individual and group sessions of mental and physical training and work-related problem solving. Pairs of RTW coordinators were in charge of coordinating and executing the on-site program for groups of maximum eight participants. Activities were organized around 6–7 h “workdays” with weekends free. Collaboration with GPs, participant work place and the social security office was initiated on-site, and participants had prepared their own action plan for RTW with guidance from on-site RTW coordinators and community stakeholders. The on-site program is described in detail elsewhere (Fimland et al., 2014).

#### Primary Outcome

The primary outcome was (re)entry to the ordinary work force analyzed from baseline and up to 1 year (56 weeks) after discharge. The primary outcome variable was dichotomous and defined as participation in competitive work ≥2.5 day (18.75 h) per week, using four different time periods with 14 weeks between each time point.

#### Independent Variable

The Metacognitions Questionnaire-30 (MCQ-30; Wells and Cartwright-Hatton, 2004) is a 30-item measure evaluating metacognitive believes across give subscales: (1) positive metacognitive beliefs about the usefulness of worry (e.g., Worrying helps me cope); (2) Negative metacognitive beliefs regarding the uncontrollability and dangerousness of worry (e.g., when I start worrying I cannot stop); (3) Beliefs about cognitive confidence (e.g., “I have a poor memory”); (4) Beliefs about the need to control thoughts (e.g., “Not being able to control my thoughts is a sign of weakness”); (5) Beliefs about cognitive self-consciousness (e.g., “I pay close attention to the way my mind works”). Items are scored from 1 to 4 (“do not agree,” “agree slightly,” “agree moderately,” “agree very much”). Cronbach’s alpha coefficients for these subscales range from 0.72 to 0.93, with test-retest correlations of: 0.75 (total score), 0.79 (positive beliefs), 0.59 (uncontrollability/danger), 0.69 (cognitive confidence), 0.74 (need for control), and 0.87 (cognitive self-consciousness) (Wells and Cartwright-Hatton, 2004).

#### Covariates

The Hospital Anxiety and Depression Scale [HADS (Zigmond and Snaith, 1983)] evaluates symptoms of *anxiety and depression.* The scale includes 14 items with two subscales: anxiety and depression. Items are scored using a four-point Likert scale ranging from 0 to 3. In a review of HADS in Norwegian adults the correlations between the two subscales varied from 0.40 to 0.74 (mean 0.56). Cronbach’s alpha for HADS-A varied from 0.68 to 0.93 (mean 0.83) and for HADS-D from 0.67 to 0.90 (mean 0.82) (Zigmond and Snaith, 1983; Bjelland et al., 2002). When investigated in the current sample, Cronbach’s alpha for the total sum score of the HADS scale had an average of 0.86, with the HADS-D having a mean Cronbach’s alpha of 0.82 and the HADS-A having a mean Cronbach’s alpha of 0.90.

The Chalder Fatigue Questionnaire [CFQ (Chalder et al., 1993)] consists of eleven questions asking about physical and mental fatigue and is frequently used to measure symptoms in chronic fatigue patients. Each item has four response categories (0–4), which are scored bi-modally 0-0-1-1. When scored, the 11 items are summed and gives each participant a score on a scale of 0–11. This eleven-item scale has been validated for a Norwegian adult population with a cut-off on symptom intensity ≥4. Cronbach’s alpha has been calculated for all items (range 0.88–0.90). Split half reliability has also been calculated (0.86 and 0.85, respectively) (Chalder et al., 1993; Loge et al., 1998). When investigated in the current sample, Cronbach’s alpha for this CFQ scale had an average of 0.86.

*Chronic pain* was measured with an item from Short Form-8 (SF-8) asking “*How much bodily pain have you had the last week*?” (None, very mild, mild, moderate, severe, and very severe). This scale has been validated as a self-report measure of chronic pain in Norwegian populations. As this is a one-item measurement, alpha values are not applicable. The item has been shown to have an intra-class correlation coefficient of 0.66 (95% CI 0.65–0.67) (Ware et al., 2001; Landmark et al., 2012).

### Statistical Methods

Descriptive statistics are used to report the participants’ baseline socio-demographic, health, psychological and work-related characteristics. *t*-tests of change on the MCQ-30 sum score are investigated as well as its subscales pre-post intervention.

Generalized estimated equations (GEE) was performed to analyze the dichotomous outcome variable (≥2.5 days of competitive work per week) using repeated measurements (RTW per 14-week period) and an unstructured working correlation structure. The variable time was treated as a categorical variable. A GEE analysis was used as it allows for the association between MCQ-30 and RTW to be estimated across several timepoints while considering the correlation between timepoints.

The first 14-week period immediately after occupational rehabilitation was used as reference category. Each 14-week follow-up period was added to the model as a as a dummy variable (i.e., post rehabilitation weeks 1–14, weeks 15–28, weeks 29–42, weeks 43–56). Precision was measured with 95% confidence intervals (CI).

To investigate the associations between RTW and change in metacognitions, each participant’s MCQ-30 total score was calculated at baseline as well as immediately after the participants completed rehabilitation, and a change score was calculated subtracting the post from the pre-value. These change scores were then used to analyze the association between change in metacognitions and probability for RTW over the four different follow-up periods.

As a sensitivity analysis to evaluate whether the observed patterns differed at different time points, interaction terms between the studied variable and each registration time-point were included in the model. Odds ratios (OR) are reported. Every GEE model was adjusted for age, gender and the underlying intervention of the randomized controlled trial. Precision was measured with 95% CI and *p* < 0.05 was considered statistically significant. Data analysis was performed using STATA version 15 (StataCorp. 2015. College Station, Texas, United States).

## Results

To be eligible for participation in the current study you had to have registry data for outcome of RTW at pre-intervention and over 56 weeks, as well as baseline (pre-intervention) data on the MCQ-30, HADS, CFQ, and SF-8. This resulted in 212 eligible participants, however, as there was a software problem during data collection, only 137 participants completed the MCQ-30 at post intervention. Our final study population consisted predominantly of females on work assessment allowance (*n* = 137). Most of the participants reported a combination of chronic pain (76.6% SF-8 > 3), fatigue (89.0% CFQ ≥ 4), and also reported mental distress (61.3% HADS > 8). Further demographics reported at baseline are presented in Table 1.

**TABLE 1:** Baseline characteristics of the study population (*n* = 137) are either presented as percentages of total N, or as mean and standard deviation (SD).

Descriptive variables	*n*/N (Mean)	% (SD)
**Socio-demographics**		
Female	112	81%
Age	43.0	SD 8.6
Higher education (high/low)	66	48%
**Work and type of benefits**		
Employed but receiving benefits	84	61%
Not currently employed	53	39%
Work assessment allowance (type of benefit >1 year)	78	56%
Sick leave (out of work <1 year)	59	44%
**Self-reported health**		
Chronic pain (SF-8 score on average pain)	3.7	SD 1.1
Chronic fatigue (Chalder fatigue scale)	8.4	SD 2.8
HADS depression	6.7	SD 4.0
HADS anxiety	7.9	SD 4.2
Sleep disturbance (ISI score)	10.3	SD 6.1
Diagnosed mental disorder (SCID)	26	19%

In order to report the absolute number of participants reaching successful outcome criteria at all the four follow-up time points, we calculated the number of participants registered as working at least 50%, averaged over a 14-week period, at the four selected follow-up time points. The raw RTW data showed that *n* = 15 (10,3%) met criteria at the first time point (14 weeks after rehabilitation), *n* = 23 (16,5%) at the second time point (28 weeks), *n* = 33 (23,7%) at the third time point (42 weeks), and *n* = 37 at the fourth time point (27,1%) (56 weeks).

In Table 2, dividing the participants into those who achieved at least 50% RTW (*n* = 39) and those who did not (*n* = 98), the baseline scores on MCQ-30 and its subscales, as well as changes from baseline to immediately after completing rehabilitation on MCQ-30 are presented.

**TABLE 2:** Averaged change on metacognitive beliefs reported by participants by those returning to work at least 50% (*n* = 39) within the 56-week period indicated as group 1, and those not meeting this criterion (*n* = 98), indicated as group 0.

Variable	Pre treatment		Post treatment		Change	Paired samples *t*-test		
	Mean	SD	Mean	SD	Mean (SD)	*t*	*p*	*g*
**Metacognitive beliefs**								
MCQ-30 total (0)	51.8	12.2	49.5	11.1	2.27 (7.8)	2.9	0.005	0.23
MCQ-30 total (1)	55.9	10.9	51.8	9.8	4.05 (7.7)	3.3	0.002	0.45
**MCQ subscales**								
Cognitive confidence (0)	12.4	4.4	11.6	4.3	0.77 (3.33)	2.3	0.030	0.24
Cognitive confidence (1)	13.3	5.0	11.9	4.5	1.38 (3.60)	2.4	0.021	0.44
Positive beliefs (0)	7.6	2.3	7.3	1.8	0.21 (1.92)	1.1	0.270	0.00
Positive beliefs (1)	7.7	1.7	7.6	1.8	0.13 (1.78)	0.4	0.655	0.00
Cognitive consci. (0)	11.4	3.5	11.2	3.5	0.05 (2.85)	0.7	0.493	0.00
Cognitive consci. (1)	12.3	3.0	12.2	3.4	0.17 (2.50)	0.1	0.911	0.00
Uncontrollability (0)	11.5	3.9	11.0	3.8	0.48 (2.89)	1.6	0.030	0.24
Uncontrollability (1)	13.0	3.9	11.7	3.1	1.33 (2.37)	3.5	0.001	0.66
Need to control (0)	9.0	2.8	8.4	2.8	0.63 (2.60)	2.4	0.018	0.50
Need to control (1)	9.7	2.5	8.5	2.4	1.15 (1.86)	3.9	<0.001	0.50

*The MCQ-30 scores are pre and post intervention. All variables were significance tested with a paired *t*-test and degree of change was described as absolute change and as a Hedges *g* effect size.*

*t*-tests of change and absolute change is reported. Paired *t*-tests indicated significant changes on metacognitions in those who returned to work, but also in the larger group not achieving RTW. On both the total sum the MCQ-30 and the subscales of cognitive confidence and beliefs about the need to control thoughts the mean change was greater in the group achieving at least 50% work. In the subscale reporting beliefs about thoughts concerning danger and uncontrollability those achieving RTW had a significant change from baseline to immediately after rehabilitation, those not achieving RTW did not (Table 2).

### Associations From the MCQ-30 Scores at Baseline

Baseline scores on the MCQ-30 were analyzed for association with RTW at all follow-up measurements spanning a year (56 weeks). None of the MCQ-30 subscales at baseline were associated with RTW at the four time points when adjusted for age, gender and the underlying intervention of the randomized controlled trial. Further details are presented in Table 3.

**TABLE 3:** Predictive associations presented as odds ratios (OR) for achieving successful 50% return to work (RTW) given metacognitions reported by participants at baseline and change in these metacognitions pre to post intervention.

*Metacognitive beliefs at baseline*	*OR (95% C.I.)*	*p-value*
*MCQ-30 total*	1.03 (1.00–1.06)	0.07
*Cognitive confidence*	1.04 (0.97–1.13)	0.27
*Positive beliefs*	1.11 (0.74–1.68)	0.59
*Cognitive consci.*	1.08 (0.98–1.19)	0.13
*Need to control*	1.12 (0.99–1.27)	0.07
*Uncontrollability*	1.07 (0.98–1.17)	0.12

***Change from pre-post intervention***	**OR (CI)**	***p*-value**

*MCQ-30 total*	1.05 (1.00–1.10)	0.04
*Cognitive confidence*	1.09 (0.99–1.21)	0.09
*Positive beliefs*	1.03 (0.78–1.36)	0.85
*Cognitive consci.*	1.00 (0.87–1.14)	0.42
*Need to control*	1.20 (1.03–1.39)	0.02
*Uncontrollability*	1.13 (0.99–1.29)	0.06

### Associations From the MCQ-30 Change Scores

Substantially higher work participation was observed for participants that reported change on the total sum of MCQ-30 from pre to post treatment. There was an association of 5% greater odds for successful RTW at all time points (*p* = 0.04) per 1-point change on the total sum of MCQ-30. On the subscale of need to control thoughts there was a 20% increase in the OR of reaching the successful outcome per 1-point change, when looking at the association over all time points (see Table 3). None of the other metacognition subscales reached statistical significance.

Sensitivity analysis: The interaction between total MCQ score and time was not statistically significant at any timepoint with reference to the first 14-week time period following rehabilitation. This was also the case for all subscales measured at baseline. Change in the subscale of beliefs about the need to control thoughts showed a significant interaction with time for the second time period 15–28 weeks (OR 0.78, CI 0.65–0.94, *p* = 0.01) and the third time period 29–42 weeks (OR 0.78, CI 0.62–0.98, *p* = 0.03) with reference to the first 14-week time period following rehabilitation.

## Discussion

The current study evaluated the prospective association between baseline metacognitions, changes in these beliefs after multi-disciplinary rehabilitation, and sustainable return-to-work over the course of 56 weeks (RTW). We did not find an association between the subscales of metacognitions or the total score of metacognitive beliefs at baseline and subsequent RTW.

However, when investigating changes in metacognitions, both a change in the total sum of metacognitions and metacognitions about the need to control thoughts substantially affected RTW. None of the interaction effects with time changed the results in a significant way, indicating that the effect from MCQ-30 on RTW is stable over time.

The results indicate that metacognitions about the need to control thoughts could be of particular interest in the work rehabilitation context. Previously published data on metacognitions and work status have shown that the need to control thoughts is significantly different in those that are working and not working when suffering from social anxiety (Nordahl and Wells, 2017b). The same substrate of metacognitions has been associated with work status above and beyond the existence of a mental disorder and trait anxiety in the same study population (Nordahl and Wells, 2017a). Thus, in combination, this lends support to the idea that metacognitive beliefs regarding the need for mental control (i.e., “not being in control of my thoughts is a weakness”) could be implicated in the ability to sustain work participation.

These specific metacognitions are thought to intensify worry about having certain thoughts, leading to enhanced monitoring or searching for threatening thoughts, which are then coupled with attempts to control metacognitive processes. It is theorized that this could increase frequency and duration of rumination and worry. If this happens, the process is debilitating for coping strategies over time, as the activation of CAS may happen as a consequence. Thus, beliefs about the need for controlling thoughts as a coping strategy is likely to have paradoxical effects such as increasing awareness of thought intrusions and using up mental capacity. This could lead to a subjective experience of cognitive dysfunction, given that a level of processing capacity is preoccupied with threat monitoring and searching interoception (Jacobsen et al., 2016). Moreover, according to the metacognitive model this could affect work capacity and drive perceptions of increased work load, ultimately enhancing negative interpretations of one’s ability to work effectively (Nordahl and Wells, 2017a).

The total score on the MCQ-30 at baseline did not predict RTW at baseline. However, the data showed that it was participants with a higher total score on MCQ-30 at baseline who subsequently reported the greatest change on the MCQ-30, and had higher odds of reaching the chosen success criteria within the follow-up period. This is an indication that participants with higher potential for change i.e., higher score on the MCQ-30, and who experience the largest change in metacognitions, are those who achieve RTW to a larger extent. This observation is in line with changes in the sum total of metacognitions predicting RTW. The observed data was supported by the GEE analysis showing that those achieving the greatest reduction in metacognitions during rehabilitation significantly increased in odds of RTW.

Previously, metacognitions about thoughts concerning danger and uncontrollability have been associated with work status (Nordahl and Wells, 2017b), and in the current study there was a trend indicating that a reduction in these metacognitions following treatment could influence RTW. When investigating RTW the deciding factor often lies in the chosen success criteria which is challenging when using longitudinal measures. In previous studies on metacognitions, the design has been cross-sectional and participant work status has been subjectively reported, which always gives a potential for misrepresentation and misunderstandings (Andersen et al., 2012). Future studies on work disability prevention programs should attempt to assess metacognitions as this may be relevant for most interventions. A larger sample size might have yielded a significant odds ratio in this study.

Another important point is that the participants’ in this study were not selected for a particular diagnosis or diagnostic category. Rather, they reflect the Norwegian population on long-term sick leave and in need of specialized occupational rehabilitation. In this population, the rule rather than the exception is comorbidity and several mental as well as physical obstacles and symptoms. A selected group of participants with common mental disorders and only mental disorders might have yielded different results. A previous publication from our group has showed the contribution of several factors when looking at prediction and facilitation of RTW and how these arguably describe different pieces of a complex puzzle (Hara et al., 2018).

The current results generate hypotheses on which factors should be designed when targeting mental obstacles when attempting to facilitate RTW. Recently there have been systematic reviews showing that adding traditional CBT in concert with RTW programs does not increase the effect of such programs above the control condition (Salomonsson et al., 2017; Cullen et al., 2018). This could in part be due to the lack of focus on metacognitions, especially those concerned with the beliefs about the need to control thoughts. We here propose that a future trial should use a randomized controlled design to evaluate an intensive RTW rehabilitation based on the metacognitive model, alongside physical therapy and RTW coordination, comparing this to an active arm using either a traditional CBT or ACT intervention.

### Limitations

A limitation to this study is the potential selection bias given the number of participants with follow-up data. There was a software problem during data collection, and only 137 participants completed the MCQ-30 at post intervention. Missing data was treated as missing completely at random (MCAR) due to no systematic drop-out. In addition, drop-out analysis demonstrated that there was an overlap between periods of non-response with reports of software and Wi-Fi-malfunctioning from the software developer. Therefore, non-response was assumed to be due to factors beyond the control of the participants. However, the use of registry data somewhat counteracts the low number of participants. It is also a limitation that the intervention used was not MCT, thus we cannot know whether an MCT targeted rehabilitation would be more adequate and yielded larger results. However, this was a secondary analysis of an RCT trial and the intervention was a result of the overarching trial.

## Conclusion

We here conclude that in participants on long-term sick leave due to chronic fatigue, pain and/or mental distress, metacognitions concerning the beliefs about the need to control thoughts appear to have a significant influence on their RTW life. Our data indicate that subtle differences in the need to control thoughts when entering rehabilitation can affect RTW. Moreover, that a reduction in the total score on MCQ-30 as well as a reduction in the need to control thoughts subscale following treatment gives significantly better odds of returning to work. We therefore recommend future studies to include these measures in RTW-rehabilitation, and propose an RCT to examine the potential effect of adding techniques from the metacognitive model to existing rehabilitation programs.

## Ethics Statement

All procedures performed in the study involving human participants were in accordance with the ethical standards of the National Research Committee and with the 1964 Helsinki declaration and its later amendments or comparable ethical standards. Written informed consent was obtained from all individual participants included in the study. The study has been approved by the Regional Committee for Medical and Health Research Ethics in Central Norway (No. 2010/2404).

## Author Contributions

HJ composed the manuscript. KH and MG performed statistical analysis. TS authored parts of the manuscript. All authors read through and commented on the manuscript.

## Conflict of Interest

The authors declare that the research was conducted in the absence of any commercial or financial relationships that could be construed as a potential conflict of interest. The reviewer BL declared a shared affiliation, with no collaboration, with one of the authors, HJ, to the handling editor at time of review.
